# The Tumor Immune Contexture of Prostate Cancer

**DOI:** 10.3389/fimmu.2019.00603

**Published:** 2019-03-28

**Authors:** Natasha Vitkin, Sarah Nersesian, David Robert Siemens, Madhuri Koti

**Affiliations:** ^1^Department of Biomedical and Molecular Sciences, Queen's University, Kingston, ON, Canada; ^2^Cancer Biology and Genetics, Queen's Cancer Research Institute, Kingston, ON, Canada; ^3^Department of Urology, Queen's University, Kingston, ON, Canada; ^4^Department of Obstetrics and Gynecology, Queen's University, Kingston, ON, Canada

**Keywords:** prostate cancer, tumor immune microenvironment (TIME), immunotherapy, immune checkpoint, DNA damage response, PTEN, hormone therapy

## Abstract

One in seven men in North America is expected to be diagnosed with prostate cancer (PCa) during their lifetime (1, 2). While a wide range of treatment options including surgery, radiation, androgen deprivation and chemotherapy have been in practice for the last few decades, there are limited treatment options for metastatic and treatment resistant disease. Immunotherapy targeting T-cell associated immune checkpoints such as CTLA-4, PD-L1, and PD-1 have not yet proven to be efficacious in PCa. Tumor mutational burden, mutations in DNA damage repair genes, immune cell composition and density in combination with their spatial organization, and expression of immune checkpoint proteins are some of the factors influencing the success of immune checkpoint inhibitor therapies. The paucity of these features in PCa potentially makes them unresponsive to contemporary immune checkpoint inhibition. In this review, we highlight the hallmark events in the PCa tumor immune microenvironment and provide insights into the current state of knowledge in this field with a focus on the role of tumor cell intrinsic events that potentially regulate immune related events and determine therapeutic outcomes. We surmise that the cumulative impact of factors such as the pre-treatment immune status, PTEN expression, DNA damage repair gene mutations, and the effects of conventionally used treatments on the anti-tumor immune response should be considered in immunotherapy trial design in PCa.

## Introduction

Prostate cancer (PCa) is the second most commonly diagnosed malignancy in men; each year, ~220,000 men in the United States are diagnosed with PCa (3). Newly diagnosed PCa is assessed using a combination of typical cancer staging (TNM), histological characteristics of a prostate biopsy, as well as prostate specific antigen (PSA) levels (4). In men diagnosed with lower risk, localized cancer, treatment options include active surveillance, radical prostatectomy (RP) or radiation therapy (RT) (4). Those with higher risk but still potentially curable disease will often require multiple interventions including RP +/- RT, as well as androgen deprivation therapy (ADT) as an adjuvant (4). However, these treatments are not curative for all patients, and biochemical recurrence occurs in approximately 25% of patients (5). Following recurrence, or for those presenting with metastatic disease, ADT is the current standard of care to remove circulating androgens that drive PCa growth and survival (6). Despite an initial clinical response, the majority of patients fail ADT and develop castration-resistant PCa (CRPC), a state of disease progression which occurs despite surgical or medical castration (7). Short term responses to systemic chemotherapy or other androgen receptor targeted therapies may occur, however, CRPC is ultimately lethal and results in the death of ~29,000 American men each year (3, 7). The high morbidity of this disease urgently necessitates the development of novel treatment strategies.

One such promising approach under investigation for PCa therapies is immunotherapeutic treatments that harness and exploit the body's intrinsic anti-tumor immune response. The recent success of immune checkpoint inhibitors (ICIs) in cancers such as melanoma and bladder cancer, has led to renewed interest in the tumor immune contexture to identify prognostic and predictive biomarkers as well as to direct novel immunotherapy combinations and sequencing toward precision cancer therapies (8). Several investigations on spatial and molecular profiling of tumors have attempted to define a pan-cancer immune landscape ranging from broad classifications as immunologically cold or hot (9), to six molecular subtypes; wound healing, interferon (IFN)-γ dominant, inflammatory, lymphocyte depleted, immunologically quiet, and transforming growth factor (TGF)-β dominant (10). Such comprehensive classification of the tumor immune microenvironment (TIME) in prostate cancer (PCa) is currently unavailable. Attributed to the disease complexity and significant heterogeneity, a deeper view of the PCa TIME is currently lacking and is needed to inform the design of immunomodulatory treatments and drug sequencing. In-depth knowledge regarding the cumulative effects of oncogenic drivers in distinct TIME states is critical to guide selection of therapies exploiting the anti-tumor immune responses. In this review, we focus on the immune features associated with localized and metastatic PCa to allow a knowledge-driven approach for future immunotherapy-based treatments.

### The PCa Tumor Immune Microenvironment (TIME)

Immune responses, involving both secreted and cellular factors in the TIME, can drastically impact the balance between tumor progression, tumor clearance, and treatment response. Specifically, the variability in response has shifted the focus of rational design of ICIs to incorporating the features of the TIME such as infiltration and localization of tumor infiltrating lymphocytes (TILs) and presence of immunosuppressive cell populations (11). Interestingly, among the genitourinary cancers, PCa exhibits a unique TIME profile with distinct features of these populations (12).

The presence of cytotoxic and helper T lymphocytes within tumor margins has been associated with favorable prognoses and clinical implications across a multitude of cancer types (13). Identifying the critical function of TILs in cancer progression led to the establishment of the “immunoscore” as a standardized metric to assess the tumor immune contexture based on the density and location of CD3+ and CD8+ T cells (13). Given that the compartmentalization of TILs within the tumor is a critical feature associated with response and outcomes, only TILs within the tumor center and invasive margins are considered in the immunoscore (14). Using this classification in combination with tumor inflammation signature, solid tumors can be broadly classified into T cell inflamed/ “hot,” and non-T cell inflamed/ “cold” tumors (15). ICI trial outcomes in some solid cancers such as melanoma urothelial and lung cancer, show that favorable responses are observed in hot tumors, which have a pre-existing higher density of TILs and expression of an IFN-associated gene signature (8, 16). Patients with an inflamed TIME also exhibit better responses to traditional therapies such as radiation and chemotherapy. Both treatment strategies are known to stimulate immunogenic cell death and consequently enhance the efficacy of immune checkpoint inhibitor therapy (14, 17).

In many solid tumors, high CD8+ TIL infiltration, especially their activated state, correlates with better prognosis due to their cytotoxic functions (18, 19). However, the prognostic relevance of CD8+ TILs is unclear in PCa, with some studies demonstrating that a high tumor TIL infiltrate is detrimental to patient survival. Indeed, one study reported that a higher density of stromal CD8+ TILs associates with poor prognosis in radical prostatectomy specimens and demonstrated a significant correlation between immunosuppressive CD73 expression and CD8+ TIL density (20). Another report showed that infiltration by CD8+ TILs within the invasive margins and stromal compartment of tumors associates with poor clinical outcomes and a shorter time until BCR in PCa patients (21). Another study evaluated tumor infiltrating CD8+ TILs and programmed death-ligand 1 (PD-L1) immune checkpoint expression in 51 node-positive PCa samples and reported that both CD8+ TIL density and PD-L1 expression were independent predictors of clinical progression (22). Most recently, an analysis of gene expression profiles of 1,567 prostatectomy specimens showed that high tumor TIL infiltrates were associated with worse distant metastasis-free survival (23). These findings may be due to improper TIL functionality; previous studies suggest that CD8+ TILs in the PCa TIME may be dysfunctional or suppressed, contributing to impaired cytotoxic responses despite tumor antigen stimulation (21, 24). It is currently unknown whether PCa-infiltrating TILs are in a state of anergy, exhaustion, or senescence; all of these are characterized by low or negligent levels of effector function (25). Further research is needed to characterize the functional status of TIL infiltrates in PCa to definitively assess the impact of their localization on prognosis.

The immune response is a balance between immunostimulatory and immunosuppressive factors; accordingly, functional TIL activity in PCa could be impaired by the magnitude of impact of secreted and cellular immunosuppressive factors. When looking at other T-cell populations in PCa, studies have noted high proportions of both CD4+ and CD8+ forkhead box P3 (Foxp3+) regulatory T cells (Tregs), within the tumor margin and epithelial compartment in PCa (26, 27). Another report examining changes in TIL infiltrates in PCa biopsies at diagnosis and subsequent relapse showed that increased infiltrates of Foxp3+ TILs were significantly associated with worse progression-free survival and overall survival (28). Preliminary data suggests that the presence of other receptors such as CCR4 on Tregs may impact PCa patient survival, although further research is required to support this claim (29). Previous reports in gastric cancer show the positive association of CD8+Foxp3+ T cells with favorable prognosis which is in contrast to findings in PCa (30). In addition to the presence of immunosuppressive lymphocytes, multiple reports have demonstrated that high tumor-associated macrophage (TAM) infiltration in the PCa TIME is pro-tumorigenic (31), however, most do not differentiate between the M1 (tumor suppressive) and M2 (tumor promoting) phenotypes of TAMs. Notably, co-culturing of naïve monocytes with PCa cells resulted in decreased expression of co-stimulatory molecules and reduced endocytic ability compared to monocytes stimulated with M-CSF (31). Furthermore, these macrophages secreted high levels of M2-associated immunosuppressive cytokines and chemokines, with TGF-β2 being the most highly expressed (31). Given the established role of TGF-β in immune exclusion, this may be one of many factors contributing to poor TIL infiltration in PCa (32). In addition to providing insights into the association between M2 macrophages and poor prognosis in PCa, the immunosuppressive role of TGF-β is critical in the context of current ICI, where targeting TGF-β prior to ICI treatment has been suggested as an approach to improve response (32).

### Factors Affecting the PCa TIME

**Figure 1:**
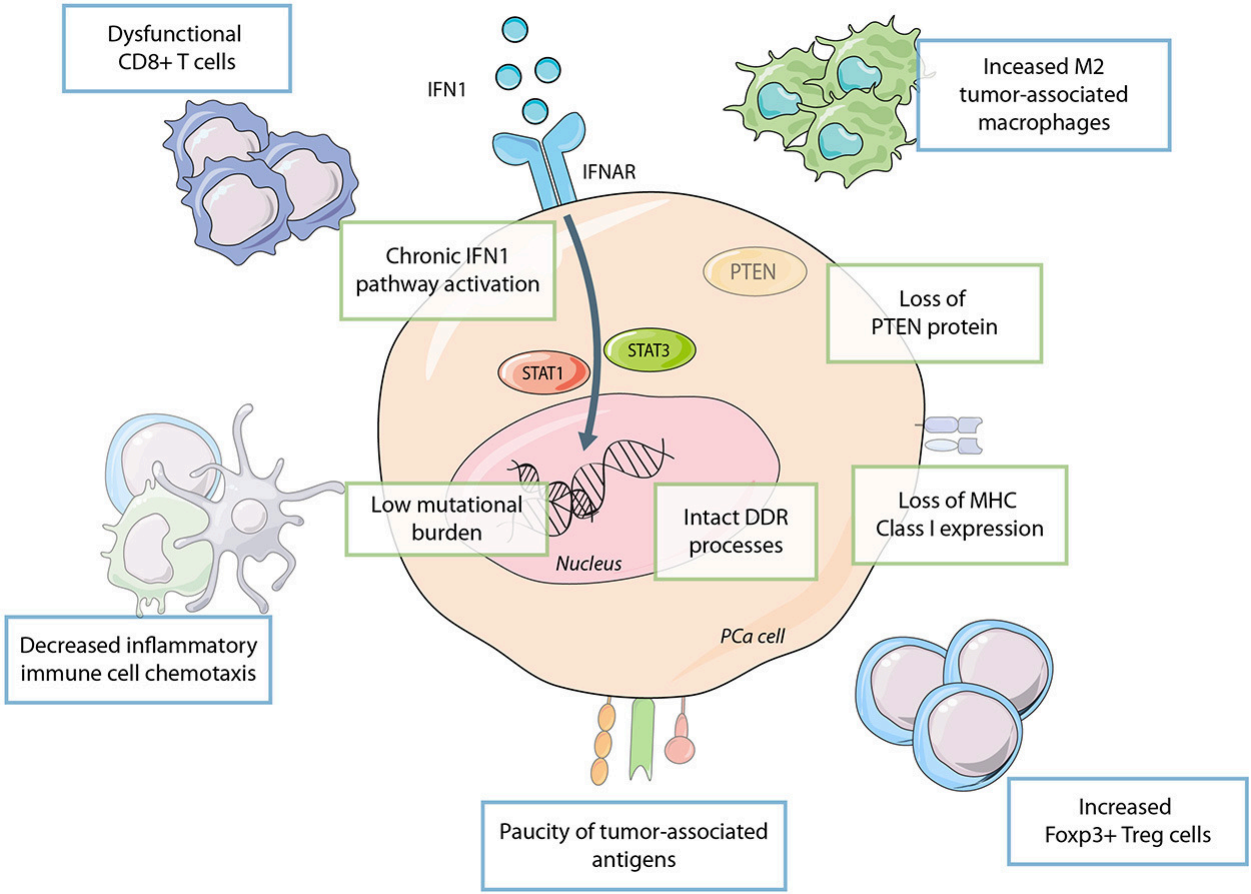
Schematic illustrating the various factors impacting the PCa TIME and their propose on the TIME. Numerous cancer cell-intrinsic factors drive the evolution of a the heterogenous pre-treatment TIME. In PCa, loss of PTEN could lead to a dysfunctional IFN1 signaling. Other features include low tumor-associated antigen expression, decreased MHC Class I expression, which could potentially contribute to immune escape. These, in combination with the pre-existing immune contexture led by other factors, are thought to be critical in determining the balance between activated and suppressive states of TILs including classical or alternatively activated tumor-associated macrophages.

The factors underlying evolution of an immunologically cold PCa TIME may be attributed to hormonal influence, genetic alterations, selective pressures of treatment. Further, immune exclusion and/or evasion mechanisms as a result of malignant progression could also lead to a cold TIME state (33). Several tumor intrinsic factors contribute to the evolution of a unique pre-treatment TIME in PCa, in addition to host physiological factors such as age and hormones. Low tumor-associated antigen expression, DDR defects, decreased MHC Class I expression, loss of PTEN protein, and dysfunctional IFN1 signaling are some of the mechanisms thought to be important in determining the features of the PCa TIME (Figure 1).

#### Tumor Mutational Burden

A feature of PCa important to the immune landscape is its relatively low somatic mutation burden and consequently diminished neoantigen expression compared to many other cancers (34). Overall rates of mutation in PCa cells are low; one study revealed a mean mutation frequency of 0.9 per megabase, about 10 times lower than that of melanoma (35). A lack of tumor neoepitopes is associated with reduced immune cell attraction to the tumor site, with fewer tumor-specific epitope-MHC interactions, resulting in reduced antigen presenting cells (APCs) cross-priming to TILs. The lack of these key interactions underlies the evolution of a non-inflamed TIME. In this scenario, transformed cells could evade immune cell-mediated elimination and proliferate freely (36). Consequently, treatment with immunotherapies would be ineffective as a pre-existing active immune contexture would be lacking. Indeed, ICI therapies such as those targeting the PD-L1/PD-1 immune checkpoint axis have the largest clinical impact in cancers with the highest numbers of somatic mutations such as melanoma and non-small cell lung cancer (37).

#### DDR Gene Defects

**Figure 2:**
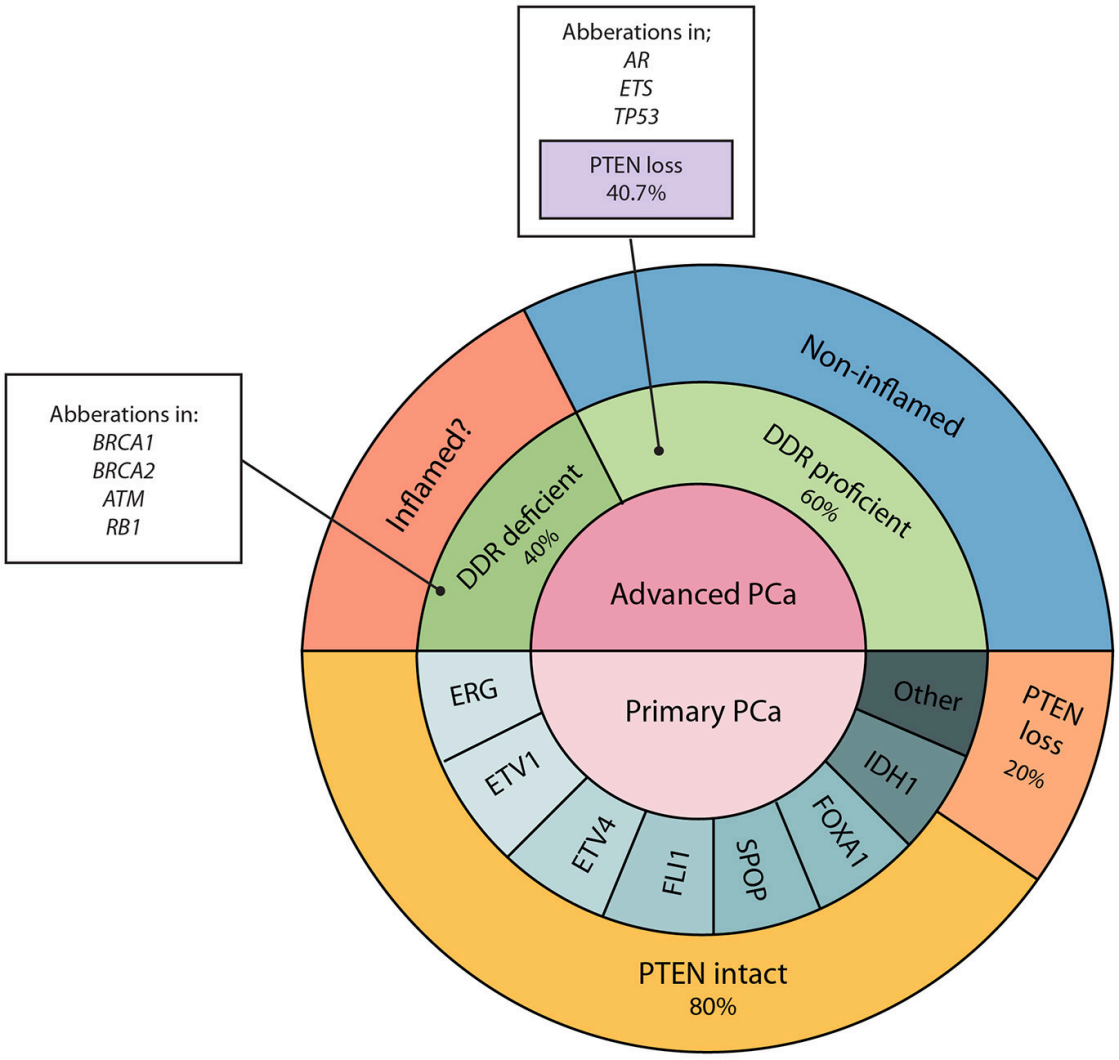
Genetic aberrations associated with primary and advanced PCa. PTEN loss is associated with 20% primary and 40.7% of advanced PCa. Increased proportions of mutations in DNA damage repair genes, *BRCA1, BRCA2, ATM*, and *RB1*, have mostly been reported in advanced PCa. While DDR deficient tumors may exhibit increased numbers of oncogenic mutations, we speculate that this may result in a more immunogenic phenotype and give rise to an inflamed TIME as observed in some other solid tumors.

DDR is an important cellular pathway initiated to drive timely and accurate repair of genetic material damaged by mutagens such as ionizing radiation. Lack of cellular DDR mechanisms can lead to the accumulation of genetic aberrations, resulting in tumor evolution and progression (38). While fostering genetic instability, these alterations are also thought to skew the TIME toward an inflamed state, partly by increasing interactions of tumor-specific antigens with infiltrating immune cells or through altering cellular IFN pathways (39). The field of DDR in PCa is relatively understudied because of its low prevalence in this cancer. However, recent next generation sequencing based profiling efforts from The Cancer Genomic Atlas Network highlight these defects in both primary and advanced PCa (40). This study, conducted on primary PCa and localized disease, showed the presence of mutations in the DDR genes *BRCA2, BRCA1, CDK12, ATM, FANCD2, RAD51C* in 19% of cases (40). Similarly, an enrichment in DDR gene mutations in the metastatic scenario was reported in 23% of cases (41). Analyses based on 150 primary and mCRPC cases showed an enrichment in aberrations in *TP53* (53%), *RB1* (21%), the *PTEN-PI3K* pathway (49%), and *AR* (63%) in mCRPC compared to localized disease (41). The presence of many molecular subtypes with different mutations in DDR pathways and driver mutations makes generalizing the TIME status in patients challenging (Figure 2).

In line with these observations, a recent trial reported that PCa patients with DDR deficiencies (*BRCA1, BRCA2*, and *ATM)* had significantly better responses to Olaparib with corresponding increases in overall survival and progression free survival (42). No differences in these metrics were reported between patients with germline mutations compared to somatic aberrations, suggesting that by the time CRPC occurs, the impacts of germline and somatic DDR defects are functionally equivalent. In localized PCa, percentage of men with germline DDR defect was lower (4.6%), and odds ratios also support a higher proportion of DDR defects in men with mCRPC compared to localized PCa (43). These results are especially promising for patients who have failed multiple treatments, as they implicate late stage PCa patients with DDR deficiency as better responders to therapy. In a study of over 600 mCRPC cases, 11.8% had a germline mutation in a prominent DDR gene, compared to only 4.6% in localized PCa patients (43). Furthermore, the presence of germline mutations in *BRCA2, ATM* and *CHEK2* were associated with histologically advanced disease (43). The challenges of mapping the primary and metastatic sites make it difficult to assign a clear trajectory of these events as secondary to treatment pressures vs. progression of an inherently aggressive cancer.

It has been established that DNA damage induces AR activity, which feeds back to activate gene expression program promoting DNA repair; both *in vitro* and *in vivo*, activating AR signaling can promote resistance to DNA-damaging agents (44). Synergistic effects of second-generation ADT and radiotherapy to decrease PCa cell survival has been shown to be mediated partly by PARP1 (45). Since recurrent PCa is treated with ADT, sensitizing tumors to radiotherapy is common, however, it may also contribute to clonal evolution and newer mutations. Regardless, as seen in other cancers (32, 46), DDR defects are indeed beneficial from an immune perspective and could potentially form the basis for immune sensitization of PCa to ICIs.

#### Loss of MHC / HLA Expression

MHC Class I proteins are normally expressed on nucleated cells and present cytosolic peptides to T lymphocytes, triggering an immunostimulatory signal cascade resulting in T cell proliferation and target cell lysis (47). Accordingly, loss of MHC Class I expression is a common immune evasion mechanism employed by a variety of cancer types (47). Defective MHC Class I may result from aberrations in multiple pathways including HLA synthesis and transport, antigen processing, or loss of critical accessory proteins (47). Preliminary evidence also suggests that epigenetic silencing of MHC Class I genes is important in PCa (48). This loss of MHC Class I expression has been documented in both metastatic PCa cell lines and clinical specimens (49, 50). Different signaling pathways including the IFN axis can also impact MHC Class I expression; in a syngeneic mouse model of PCa, treatment with IFN-γ led to increased survival and heightened expression of proteins important in MHC Class I production such as TAP1 (51). Cell line experiments have also demonstrated that radiation increases MHC Class I expression and leads to unique MHC Class I binding antigenic peptides (52). Increased MHC Class I expression in tumors is predicted to facilitate the activation and expansion of CD8+ TILs within the invasive margins of the tumor, eliciting a more robust immune response. However, in the context of an immunosuppressive TIME lacking a dense TIL infiltrate, heightened expression of MHC Class I proteins in isolation is unlikely to shift the TIME toward an immunoactive state, especially in cases with concurrent immunosuppressive features.

#### PTEN Loss

A well-characterized molecular aberration in PCa is the loss of the tumor suppressor protein PTEN. PTEN is generally known as a lipid and protein phosphatase encoded by the *PTEN* gene which antagonizes the pro-growth PI3K signaling pathway and is deleted in up to 30% and mutated in 2-5% of primary PCa cases (53). Emerging literature suggests that the immune regulatory functions of PTEN are mediated through modulating the activation of cellular IFN1 pathways (54). In other cancers such as melanoma, patients with PTEN loss exhibited significantly poorer responses to PD-1 ICI and had lower TIL infiltration compared to patients with >10% of tumor cells positive for PTEN staining (55). Furthermore, the therapeutic activity of tumor-specific TILs from adoptive T cell therapy was significantly reduced in mice with *PTEN-*silenced melanoma cells compared to those with an intact *PTEN* gene, indicating that PTEN can confer sensitivity to T-cell-based immunotherapy (55). Other alterations may also cooperate with PTEN loss to drive distinct tumor immunological phenotypes. Using *in vivo* models, a recent study demonstrated the qualitative and quantitative impact of *Pten* loss in the TIME. Specifically, myeloid-derived suppressor cell (MDSC) infiltrates in *Pten*^−/−^*; Zbtb7a*^−/−^ prostate tumors exhibited a distinct phenotype affecting NF-κB signaling whereas MDSCs within *Pten*^−/−^*; Tp53*^−/−^ tumors were associated with Treg immunosuppression (56). These findings implicate PTEN as a tumor suppressor which, in addition to regulating the PI3K-Akt-mTOR signaling network, can govern the tumor immune milieu and response to immunotherapy, however, these findings must be validated in PCa. These data provide compelling evidence for an undefined mechanistic role of PTEN in altering the immune contexture of the PCa TIME. Recent studies conducted in phosphatase inactive PTEN cells have highlighted its phosphatase independent tumor suppressive functions, specifically in DNA repair and apoptosis (57, 58). An area relatively understudied in PCa, however, is the specific effect of altered levels of nuclear, cytoplasmic and secreted PTEN proteins in mediating an aggressive disease and immunosuppressed TIME state. Given that all three isoforms of PTEN exert different regulatory functions (59), in processes that alter cancer progression and immune response in the TIME, future investigations should incorporate these in scenarios where PTEN deficiency is not attributed to loss of 10q region harboring the *PTEN* gene. A precise definition of these genotype and associated immunophenotype relationships will allow the development of alternate targeted therapies and improved patient stratification.

#### IFN1 Signaling

Few studies have characterized the functional status of immune cell populations in the PCa TIME, but preclinical and clinical data supports that IFN1 signaling in the TIME exerts protective anti-tumor effects in PTEN-deficient tumors. IFN1 is an important group of immunostimulatory cytokines released in response to direct binding of IFN1 to its extracellular receptor, or from cellular detection of invading pathogens by innate pattern recognition receptors (60). It is established that IFN1 is crucial to mounting an efficient anti-tumor immune response, which is accomplished by a variety of mechanisms such as cytokine and chemokine production, increasing the expression of immune costimulatory molecules, activating adaptive immune cells, and facilitating CTL killing (61). The activation of transcription factors STAT1 and STAT3 drive canonical IFN1 signaling by mediating the transcription of over 2000 interferon-stimulated genes, which serve a diverse array of functions involved in stimulating and regulating the innate and adaptive immune responses (62). Combined prostate-specific *STAT3* and *PTEN* deficient mice exhibited accelerated cancer progression and metastasis compared to *PTEN-*deficient mice; these animals had tumors up to six times larger than *PTEN*^−/−^ mice (63). The authors show that these effects are mediated through the ARF-MDM2-p53 axis, and suggest that PTEN-deficient tumors cannot effectively activate this axis, resulting in tumor metastasis (63). However, conflicting evidence has demonstrated that STAT3 inhibition results in decreased PCa cell growth and tumor metastasis, both *in vitro* and animal models of PCa (64, 65). Chronic IFN1 signaling has been associated with immunosuppression and therapy resistance; both unphosphorylated STAT1 and STAT3 (U-STAT1/3) can serve as active transcription factors and mediate the expression of specific subsets of ISGs (66, 67). The subset of ISGs activated by U-STAT1 after prolonged IFN1 exposure render cancer cells insensitive to radiation and chemotherapy (68) Multiple studies have demonstrated that in addition to contributing to therapeutic resistance, these genes also promote cancer growth and metastasis (69). The ability of IFN1s to modulate the expression of distinct sets of ISGs through differences in signal duration and STAT activation provides a mechanism to account for the opposing roles of IFN1 in immune stimulation and regulation. Furthermore, it is likely that cellular and environmental cues such as PTEN loss, DDR defects, TIL infiltration and activity, and the presence of immunosuppressive factors reflect these divergent findings.

#### Impact of Therapy on the PCa TIME

Androgens and their receptors play a critical role in both progression and treatment of PCa. Antagonists of androgen receptor (AR) such as bicalutamide and enzalutamide are therefore widely used as part of ADT therapy in PCa (70). As the immune response is a dynamic process affected by environmental factors, PCa treatments can also affect the tumor immune contexture. Complex mechanisms of androgen blockade mediated effects on the PCa TIME, ranging from thymic enlargement, increased lymphocyte migration, to GABA-A receptor mediated off-target effects leading to impaired T cell priming have been reported (71). Due to the dependency of PCa cells on androgen signaling, ADT treatment results in cancer cell apoptosis, failing to release immunostimulatory signals (72). In a syngeneic murine model, increased CD3+ T cell infiltration in tumors post orchiectomy (surgical castration) with corresponding tumor regression was observed, albeit eventual relapse (71). This response was associated with a thymic T cell wave, which is typically short-lived, and may be accompanied by increases in regulatory immune cell populations (73). Suppression of both cell mediated and humoral immune responses by AR antagonists (medical castration) has been reported in syngeneic murine models of PCa (74, 75). A key finding is the contrasting impact of medical vs. surgical castration on T cell priming, which is a critical factor in anti-tumor immune response. While treatment with gonadotrophin-releasing hormone analogs has similar effects as orchiectomy, opposite effects were observed using AR antagonists. Clearly, more longitudinal studies in patients are warranted to define these precise correlations for effective sequencing of AR antagonists and immune based therapies. Similarly, given their predictive importance (76), and expression of PD-L1, defining the TAM phenotypes that associated with pre- and post ADT treated tumors will be crucial for determining the proper sequencing of ICI treatment. Another important question that remains unanswered pertains to how these changes correlate with the pre-treatment TIME states, specifically with regard to stromal and epithelial localization of cytotoxic TILs.

Treatment-induced ICD leads to the release of cancer cell antigens to which the immune system can respond (77). This mediates the influx and activation of dendritic cells (DCs) and TILs, which can facilitate a more robust anti-tumor immune response. Notably, the presence of an active immune contexture predicts a favorable response to chemotherapy, implicating that cells of the TIME are critical for an individual's response to treatment (72, 78). Docetaxel, an effective systemic chemotherapy used for men with metastatic CRPC, does not initiate classic ICD although studies suggest that it can augment TIL-mediated tumor killing and decrease MDSC populations (79, 80). In a Phase II clinical trial, metastatic CRPC patients receiving a PSA vaccine and subsequent docetaxel had a median progression-free survival of 6.1 months while patients taking docetaxel alone survived 3.7 months (81). These results suggest that while not directly inducing ICD, docetaxel treatment for CRPC patients may potentiate the immune response and mediate an inflamed TIME.

Radiation therapy is another therapeutic modality, utilized for both curative and palliative indications, that also has been demonstrated to have immunomodulatory properties. Radiotherapy has been shown to increase the number and diversity of tumor-specific surface peptides and expression of MHC Class I molecules in a dose-dependent manner, which increased the efficacy of TIL-mediated cancer cell killing (52). Immuno-potentiation may also be attributed to the release of immunostimulatory cytokines and danger-associated molecular patterns (DAMPs) due to radiation exposure (73). The abscopal effects of radiation on distant metastases in PCa have also been documented; metastatic patients who received first-line radiotherapy had significantly higher overall survival compared to patients who did not receive this treatment in one retrospective study (82). It could be hypothesized that these outcomes could be secondary to radiotherapy-instigated immune activation, which would mediate a systemic anti-tumor immune response targeting distant metastases as well as the primary tumor.

Another relatively understudied area in PCa is the difference in TIME profiles in primary tumors compared to metastatic disease. A recent landmark study comparing 150 matched primary and metastatic CRPC reported novel clinically actionable aberrations, including higher frequencies of aberrations in DDR genes such as *BRCA1, BRCA2*, and *ATM* (41). Given the availability of tumor molecular profiles from immunologically distinct sites of metastasis in studies such as this, a comprehensive characterization of the spatial and molecular immune profiles of metastatic lesions could provide an improved understanding of immune evasion mechanisms in PCa.

### Current State of Immunotherapy in PCa

Two vaccine-based immunotherapy approaches have shown moderate success in PCa treatment. Sipuleucel-T is a personalized treatment constituting the *ex vivo* expansion and activation of patient-derived peripheral blood mononuclear cells (PBMCs) with a recombinant prostate-specific fusion protein (83). The registration trial involved CRPC patients and those receiving this treatment had in a median survival was 4.1 months longer than placebo-treated patients (83). Other additional immunotherapeutic approaches, including several vaccine trials including GVAX, and PROSTVAC, however did not demonstrate a survival benefit compared to placebo in phase 3 trials despite encouraging early results (84–86).

ICI treatment in PCa has to date demonstrated less than exciting results; a Phase III trial testing CTLA-4 blockade (Ipilimumab) did not observe any differences in overall survival compared to placebo in CRPC patients (87). Ipilimumab, analyzed in two Phase III studies, did not show any survival benefit in this tumor. The KEYNOTE-199 study analyzed the role of pembrolizumab for post-docetaxel mCRPC patients and concluded that pembrolizumab had antitumor activity and acceptable safety in these patients (88). Its activity was observed both in PD-L1 positive and PD-L1 negative cohorts, however, the response rate was low, with a complete and partial response of <5% (88). To date, immune checkpoint inhibitors have yet to be FDA-approved for the management of metastatic PCa (86).

These and other data suggest that ICI alone may not be enough to facilitate a robust anti-tumor immune response in PCa patients, rather, activating tumor-specific TILs may provide more benefit. Future clinical trials investigating these agents should be encouraged on specific patient subsets including those with high PD-L1 expression, those with hypermutated or microsatellite-unstable tumors, and those enriched for germline and/or somatic DNA-repair gene mutations (e.g., intraductal/ductal histology, primary Gleason pattern 5, and perhaps AR-V7-positive tumors). Furthermore, neoadjuvant treatments which promote the development of an immunoreactive TIME could increase the sensitivity of CRPC patients to ICI and immunotherapy.

As the PCa TIME is usually non-inflamed and dominated by immunosuppressive cells, targeting or reprogramming these suppressive cell populations could skew the PCa TIME toward an inflamed phenotype and make PCa amenable for immunotherapy treatments. Accordingly, neoadjuvant administration of IFN1 agonists which activate cytosolic innate immune sensing pathways such as those mediated TLRs or STING, represents an area of unrealized potential in immunotherapy research for PCa. Preclinical findings have been promising; for example, the addition of intra-tumoral STING agonist injection to combination ICI treatment in a syngeneic mouse model of PCa increased overall survival by 35% compared to combination ICI alone (89). In this study, mice treated with both STING agonist and combination ICI had increased TIL: Treg and TIL: macrophage proportions, and decreased percentages of TAMs (89). Furthermore, it was demonstrated that this activation was not limited to STING agonists; poly I:C treatment in a syngeneic PCa mouse model has also shown to increase cellular differentiation and promote immunologically active lymphocyte infiltration (90). A more comprehensive understanding of the factors conferring sensitivity to IFN1 agonists is warranted as this approach moves forward. Discerning the immune pathways and mechanisms which significantly contribute to causing an inflamed and immunologically active TIME is required before these pathways can be therapeutically exploited. Finally, more trials, such as the recently initiated Quick efficacy seeking trial (Quest1) (86), are needed to determine precise immunotherapy combinations in PCa.

## Conclusions and Future Perspectives

A detailed analysis of treatment naïve and treatment associated TIME is not currently available in PCa with reports to date mainly focusing on evaluation of limited phenotypes of activated or dysfunctional immune cell types. Sex-steroids, primarily androgens, play important roles in thymic involution or rejuvenation and thus therapeutic ablation of these could have significant impacts on the PCa TIME. The unique clinical and molecular features of each PCa case make it difficult to predict the status of the TIME, although some metrics such as TGFβ signaling and Treg infiltration may be useful. Importantly, use of genetic alterations such as PTEN loss and DDR status should be incorporated in trial design and accompany retrospective and prospective immune monitoring correlative studies.

## Author Contributions

MK and DS conceptualized the review. NV, SN, MK, and DS wrote and reviewed the review. NV and SN generated the figures.

### Conflict of Interest Statement

The authors declare that the research was conducted in the absence of any commercial or financial relationships that could be construed as a potential conflict of interest.
